# Neutrophil Expression of Decay Accelerating Factor, a Key Complement Regulator, Has No Impact on Acute Kidney Injury

**DOI:** 10.34067/KID.0000000890

**Published:** 2025-06-10

**Authors:** Barbara Franchin, Paolo Cravedi

**Affiliations:** Renal Division, Department of Medicine, Translational Transplant Research Center (TTRC), Icahn School of Medicine at Mount Sinai, New York, New York

**Keywords:** AKI, complement

AKI is a serious clinical condition affecting approximately 10%–15% of hospitalized individuals and up to 50% of critically ill patients in intensive care units. Increasing evidence highlights the role of the immune system in AKI onset and progression. Renal neutrophil infiltration is a common feature in mouse models of AKI model and in humans and correlates with disease severity.^1^ Neutrophils transmigrate from the circulation into the kidney interstitium, where they release proinflammatory molecules and neutrophil extracellular traps that induce tubular epithelial cell injury and death, endothelial dysfunction, and fuel further infiltration of immune cells.^2^

In aristolochic acid (AA)-induced AKI, neutrophil infiltration occurs early and serves as a critical marker of tubular injury, as demonstrated by studies using neutrophil depletion strategies.^2^

Complement is strongly linked to neutrophil migration and activation in peripheral tissues, and blockade of C3a receptor ameliorates the severity of AA-induced AKI.^3,4^ Of note, decay accelerating factor (DAF or CD55) represents a key regulator of complement activation on cell membranes.

To study the role of DAF on neutrophil activation and AKI severity, we administered AA (5 mg/kg, Sigma-Aldrich, 102501333) i.p. every other day for 5 days (day 1, 3 and 5) to 8-week-old C57BL/6J (B6, H-2b) male mice that were euthanize at 14 days after the first injection. Kidneys were processed to obtain single-cell suspensions (MACS dissociation kit, Miltenyi Biotec) for flow cytometric analyses.

When euthanized, AA-injected mice showed significantly more CD11b^+^Ly6g^+^ neutrophils in the kidneys than control, PBS-injected mice (Figure 1A). We next tested changes in DAF expression in neutrophils over time after AA injection (Figure 1B). We found a stable decrease in DAF expression in neutrophils infiltrating the kidneys, while DAF expression in splenic neutrophils initially raised on day 1 after AA followed by a reduction (Figure 1C), suggesting a mechanistic link among changes in DAF expression, neutrophil recruitment into the kidney, and renal injury (Supplemental Methods).

**Figure 1:**
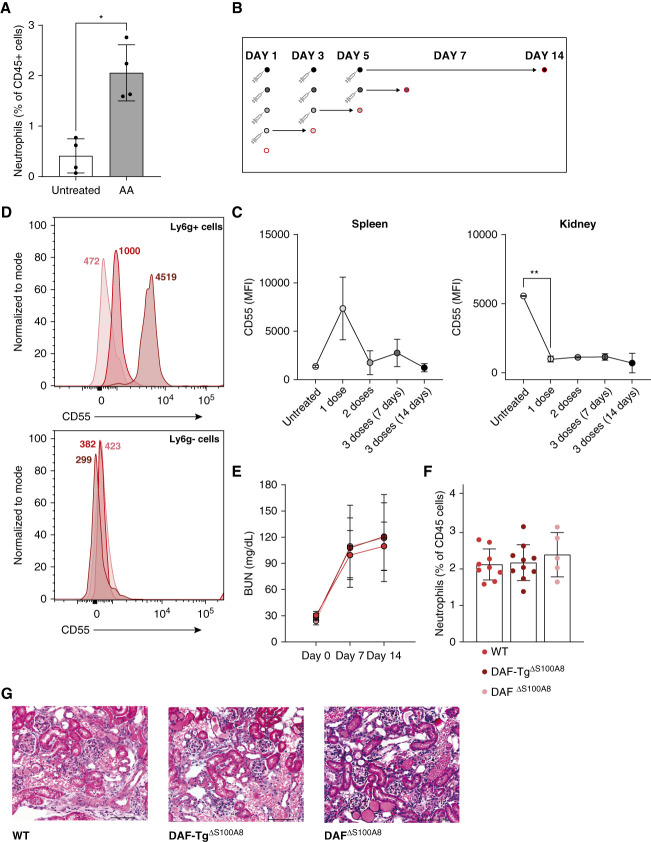
**DAF expression levels in neutrophils do not affect AA-induced AKI in mice.** (A) CD45^+^CD11b^+^Ly6g^+^Ly6c^−^ neutrophils in the kidneys of WT mice at 14 days after AA (*n*=4) or vehicle (*n*=4) injection. (B and C) WT mice received three AA injections and were euthanized at serial time points (study design) to measure the MFI of CD55 expression in CD45^+^CD11b^+^Ly6g^+^Ly6c^−^ neutrophils in spleen and kidney tissue (*n*=3 per time point). (D) Representative plots of CD55 expression in CD45^+^CD11b^+^Ly6g^+^Ly6c^−^ neutrophils (top) and in CD45^+^CD11b^+^Ly6g^−^Ly6c^+^ macrophages (bottom) from WT (*n*=9), DAF-Tg^ΔS100A8^ (*n*=10), and DAF^ΔS100A8^ (*n*=5) mice. Numbers in the two plots represent MFI. (E) Serial BUN levels at 0, 7, and 14 days in WT (*n*=9), DAF-Tg^ΔS100A8^ (*n*=10), and DAF^ΔS100A8^ (*n*=5) mice after AA injection. (F) Kidney neutrophil infiltration at 14 days after AA injection. (G) Representative kidney sections at 14 days after AA injection in WT, DAF-Tg^ΔS100A8^ and DAF^ΔS100A8^ mice (hematoxylin and eosin staining). Acquisition of images was performed on the wide-field microscope (Zeiss AxioImager Z2M). *t* Test or ANOVA: *P < 0.05; **P < 0.01. AA, aristolochic acid; DAF, decay accelerating factor; MFI, mean fluorescence intensity; WT, wildtype.

To formally test this hypothesis, we generated B6 mice that selectively lack (DAF^ΔS100A8^) or overexpress (DAF-Tg^ΔS100A8^) *DAF* in neutrophils.

First, we verified that DAF expression was selectively modified in neutrophils (Figure 1D). We also found no significant differences in the number of Ly6g^+^ neutrophils in the spleen or in the kidneys of DAF^ΔS100A8^ or DAF-Tg^ΔS100A8^ mice at baseline (not shown).

Next, we compared the severity of kidney injury upon AA injection across the groups. Contrary to our prediction, BUN levels at 7 and 14 days after AA injection did not differ among the three groups (Figure 1E). Kidney neutrophil infiltration (Figure 1F) and histological lesions (Figure 1G) were also similar. Overall, these data suggest that DAF expression on neutrophils does not affect their renal recruitment or the severity of AKI in mice.

DAF is a key regulator of local complement activation and has been proposed as a therapeutic target to control complement activation locally. Prior studies by our group and others have demonstrated the critical role of DAF in glomerular diseases, where it limits complement activation and the generation of C3a and C5a, thereby modulating signaling through their respective receptors on glomerular cells.^5^ C5a/C5aR1 signaling in neutrophils is critical for their extravasation in vasculitides, and C5aR1 blockade is used clinically to prevent disease relapse in patients with ANCA-associated vasculitis.^6^ Therefore, we hypothesized that DAF overexpression would mitigate disease severity by restricting local complement activation on neutrophils, whereas conditional DAF deletion would exacerbate it.

The present findings, although unexpected, align with the observation that the deletion of C5aR1 in myeloid cells (including neutrophils) does not affect AA-induced AKI. Notably, selective deletion of C5aR1 in tubular cells in this model results in detrimental effects rather than protective ones, as C5aR1 signaling restricts excessive glycolysis and supports mitochondrial function in tubular cells.^7^

It is conceivable that DAF expression in neutrophils may influence the severity of renal injury in other AKI models, such as ischemia-reperfusion injury. In addition, the S100A8 gene, which was used for the conditional deletion or overexpression of DAF, is not exclusive to neutrophils but also includes macrophage progenitors.^8^ Although we did not detect off-target expression of DAF deletion or overexpression, it is not possible to formally exclude that DAF has different, theoretically opposite effects on different cells. However, this seems unlikely, because both downregulation and overexpression of DAF in S100A8+ cells had no impact in the AA-induced AKI model.

Overall, these results contribute to the growing body of evidence indicating that DAF and complement have distinct and possibly phase-dependent roles in different immune and renal cell populations during AKI. A deeper understanding of complement and its regulators is crucial for the optimal application of the expanding array of complement-targeting therapies.

## Supplementary Material

## Data Availability

There are no data underlying this work.
