# Supplementary material for: Neutrophil Expression of Decay Accelerating Factor, a Key Complement Regulator, Has No Impact on Acute Kidney Injury
Source: Kidney360. 2025 Jun 10;6(10):1790–2. doi: 10.34067/KID.0000000890 (PMC12778023; doi:10.34067/KID.0000000890)
Supplement: Supplementary file 1 [file kidney360-6-1790-s001.pdf]

ASN American Society of Nephrology | JASN Journal of the American Society of Nephrology | KIDNEY360 Accessing Our World From Every Angle | CJASN Clinical Journal of the American Society of Nephrology

ASN Journal Disclosure Form

As per ASN journal policy, I have disclosed any financial relationships or commitments I have held in the past 36 months as included below. I have listed my Current Employer below to indicate there is a relationship requiring disclosure. If no relationship exists, my Current Employer is not listed.

P. Cravedi reports the following:
Consultancy: Chinook Therapeutics.; Calliditas Therapeutics.; Cerium Pharmaceuticals; Apellis; Borealis; Research Funding: Renel Research Institute; Borealis; Honoraria: Advisor for Borealis Therapeutics and Apellis.; and Advisory or Leadership Role: Associate Editor for Journal of Nephrology (JN) and American Journal of Transplantation (AJT).;.

I understand that the information above will be published within the journal article, if accepted, and that failure to comply and/or to accurately and completely report the potential financial conflicts of interest could lead to the following: 1) Prior to publication, article rejection, or 2) Post-publication, sanctions ranging from, but not limited to, issuing a correction, reporting the inaccurate information to the authors' institution, banning authors from submitting work to ASN journals for varying lengths of time, and/or retraction of the published work.

Name: Paolo Cravedi
Manuscript ID: K360-2025-000444R1
Manuscript Title: Neutrophils Expression of Decay Accelerating Factor (DAF), a Key Complement Regulator, has no Impact on Acute Kidney Injury
Date of Completion: May 19, 2025
Disclosure Updated Date: May 19, 2025

ASN Journal Disclosure Form

As per ASN journal policy, I have disclosed any financial relationships or commitments I have held in the past 36 months as included below. I have listed my Current Employer below to indicate there is a relationship requiring disclosure. If no relationship exists, my Current Employer is not listed.

B. Franchin has nothing to disclose.

I understand that the information above will be published within the journal article, if accepted, and that failure to comply and/or to accurately and completely report the potential financial conflicts of interest could lead to the following: 1) Prior to publication, article rejection, or 2) Post-publication, sanctions ranging from, but not limited to, issuing a correction, reporting the inaccurate information to the authors' institution, banning authors from submitting work to ASN journals for varying lengths of time, and/or retraction of the published work.

Name: Barbara Franchin
Manuscript ID: K360-2025-000444R1
Manuscript Title: Neutrophils Expression of Decay Accelerating Factor (DAF), a Key Complement Regulator, has no Impact on Acute Kidney Injury
Date of Completion: May 19, 2025
Disclosure Updated Date: May 19, 2025
