# Supplementary material for: Neutrophil Expression of Decay Accelerating Factor, a Key Complement Regulator, Has No Impact on Acute Kidney Injury
Source: Kidney360. 2025 Jun 10;6(10):1790–2. doi: 10.34067/KID.0000000890 (PMC12778023; doi:10.34067/KID.0000000890)
Supplement: Supplementary file 2 [file kidney360-6-1790-s002.pdf]

SUPPLEMENTAL METHODS

Table of contents

Mice

Flow cytometry

Mice

C57BL/6J (B6) were purchased from The Jackson Laboratory.

To generate DAF$^{\Delta S100A8}$ mice, we obtained embryonic stem cells from the European Conditional Mouse Mutagenesis Program (EUCOMM), into which loxP sites were inserted flanking exons 3 and 4 of the *Cd55* (Daf1) gene. The embryonic stem cells were injected into pseudopregnant B6 mice (The Jackson Laboratory) by standard techniques at the Mouse Genetics and Gene Targeting CoRE facility at Icahn School of Medicine at Mount Sinai. Founders were validated by genotyping and then crossed with B6 S100A8-cre mice (The Jackson Laboratory).

Generation of conditional DAF transgenic mice (DAF-Tg $^{\Delta S100A8}$) was performed using the coding sequence of the mouse *Cd55* gene containing the complement regulatory domain, replacing the signal sequence for glycophosphatidylinositol-(GPI)-anchor addition with that of the transmembrane helix domain of human tissue factor. Purified plasmid DNA constructs from transformed *E. coli* were verified by sequencing, transferred into fertilized DBA2/B6 F1 hybrid eggs by pronuclear microinjection and injected into pseudopregnant animals at the Mouse Genomics Core Facility (Mount Sinai). Founders were validated by genotyping and then were backcrossed to C57BL/6J. Next, DAF-Tg mice were crossed with B6 S100A8-cre mice. In these mice, transgene expression is driven by the CAG promoter in the S100A8 locus.

All animals were housed in the Center for Comparative Medicine and Surgery at the Icahn School of Medicine at Mount Sinai under Institutional Animal Care in accordance with guidelines of the Association for Assessment and Accreditation of Laboratory Animal Care International. Animal study protocols were approved by the institutional animal care and use committee at Icahn School of Medicine at Mount Sinai (New York, NY; IACUC ID PROTO202000107). The study protocol was not registered elsewhere. The sample size was not based on a formal power analysis. No animals were excluded from the analyses.

Flow cytometry

Flow cytometry surface staining was performed with: CD45 (clone: 30-F11, catalog: 11-0451-82; Invitrogen), CD11b (clone: M1/70, catalog: 45-0112-82; Invitrogen) Ly6c (clone: HK1.4, catalog: 17-5932-82; Invitrogen), Ly6g (clone: 1A8, catalog: MBS37818; MyBioSource), CD55 (clone: RICO-5, catalog: 558037; BD Biosceiences).

Data were acquired on a three-laser Canto II flow cytometer (BD Biosciences) and analyzed using FlowJo (https://www.flowjo.com) software.
